# The long noncoding RNA RNCR2 directs mouse retinal cell specification

**DOI:** 10.1186/1471-213X-10-49

**Published:** 2010-05-11

**Authors:** Nicole A Rapicavoli, Erin M Poth, Seth Blackshaw

**Affiliations:** 1 Department of Neuroscience, Neurology and Ophthalmology, Center for High-Throughput Biology and Institute for Cell Engineering, Johns Hopkins University School of Medicine, 733 N. Broadway Avenue, Baltimore, MD USA

## Abstract

**Background:**

Recent work has identified that many long mRNA-like noncoding RNAs (lncRNAs) are expressed in the developing nervous system. Despite their abundance, the function of these ncRNAs has remained largely unexplored. We have investigated the highly abundant lncRNA RNCR2 in regulation of mouse retinal cell differentiation.

**Results:**

We find that the RNCR2 is selectively expressed in a subset of both mitotic progenitors and postmitotic retinal precursor cells. ShRNA-mediated knockdown of RNCR2 results in an increase of both amacrine cells and Müller glia, indicating a role for this lncRNA in regulating retinal cell fate specification. We further report that RNCR2 RNA, which is normally nuclear-retained, can be exported from the nucleus when fused to an IRES-GFP sequence. Overexpression of RNCR2-IRES-GFP phenocopies the effects of shRNA-mediated knockdown of RNCR2, implying that forced mislocalization of RNCR2 induces a dominant-negative phenotype. Finally, we use the IRES-GFP fusion approach to identify specific domains of RNCR2 that are required for repressing both amacrine and Müller glial differentiation.

**Conclusion:**

These data demonstrate that the lncRNA RNCR2 plays a critical role in regulating mammalian retinal cell fate specification. Furthermore, we present a novel approach for generating dominant-negative constructs of lncRNAs, which may be generally useful in the functional analysis of this class of molecules.

## Background

Recent studies have demonstrated that non-protein coding RNAs (ncRNAs) comprise much of the mammalian transcriptome [1]. Several thousand mammalian ncRNAs have been identified that span multiple kilobases in length [2]. Some of these ncRNAs show extensive conservation at the nucleotide level, despite lacking evolutionarily conserved open reading frames (ORFs). Many also show characteristics reminiscent of protein-coding mRNAs, such as splicing, transcription by RNA Polymerase II, polyadenylation, and 5'capping [3].

Perhaps the best characterized mRNA-like ncRNAs are the Xist/Tsix transcripts, which mediate X-inactivation in placental mammals [4]. Other long ncRNAs such as Air and H19 have been implicated in genomic imprinting [5,6]. In both of these cases, ncRNAs act locally, coating the nearby genomic loci and inducing the formation of heterochromatin and repression of gene expression [7]. Recent studies have identified other long ncRNAs that regulate transcription at loci on different chromosomes [8]. Some of these ncRNAs may function as RNA-based transcriptional coregulators [9,10]. Other vertebrate long ncRNAs have been implicated in the regulation of protein translation [11] and signal transduction [12] and they are likely to fulfill diverse cellular functions.

Previous work has shown that many long ncRNAs are selectively expressed in the developing nervous system [13]. No functional data on their role in neuronal development is available for the vast majority of neuronally-expressed lncRNAs, however. Two notable exceptions are Evf-2, which regulates Dlx2-dependent activation of transcription of Dlx6 and development of GABAergic neurons [14,15], and Tug1, which regulates retinal rod photoreceptor development and survival [16]. The developing retina, in particular, expresses a diverse assortment of lncRNAs. Many of these are transcribed in an a divergent, head-to-head fashion with over one third of retinally-expressed transcription factors [17]. Other classes of lncRNAs, which are not located near protein-coding genes, are also strongly expressed in the retina. The most abundant of these, which represents nearly 0.2% of all polyadenylated RNA in the neonatal retina was first described as Retinal Non-coding RNA 2 (RNCR2), also known as both Gomafu and Miat [18-20]. This 9 kb ncRNA is expressed widely in the developing nervous system in an uncharacterized subset of neural precursor cells and is targeted to an as yet uncharacterized domain of the nucleus. In this study, we perform a detailed analysis of the cellular expression pattern and the function of RNCR2 in retinal differentiation. In addition, we devised an experimental strategy to develop dominant-negative forms of RNCR2.

## Results

### Evolutionary conservation and genomic organization of RNCR2

**Figure 1:**
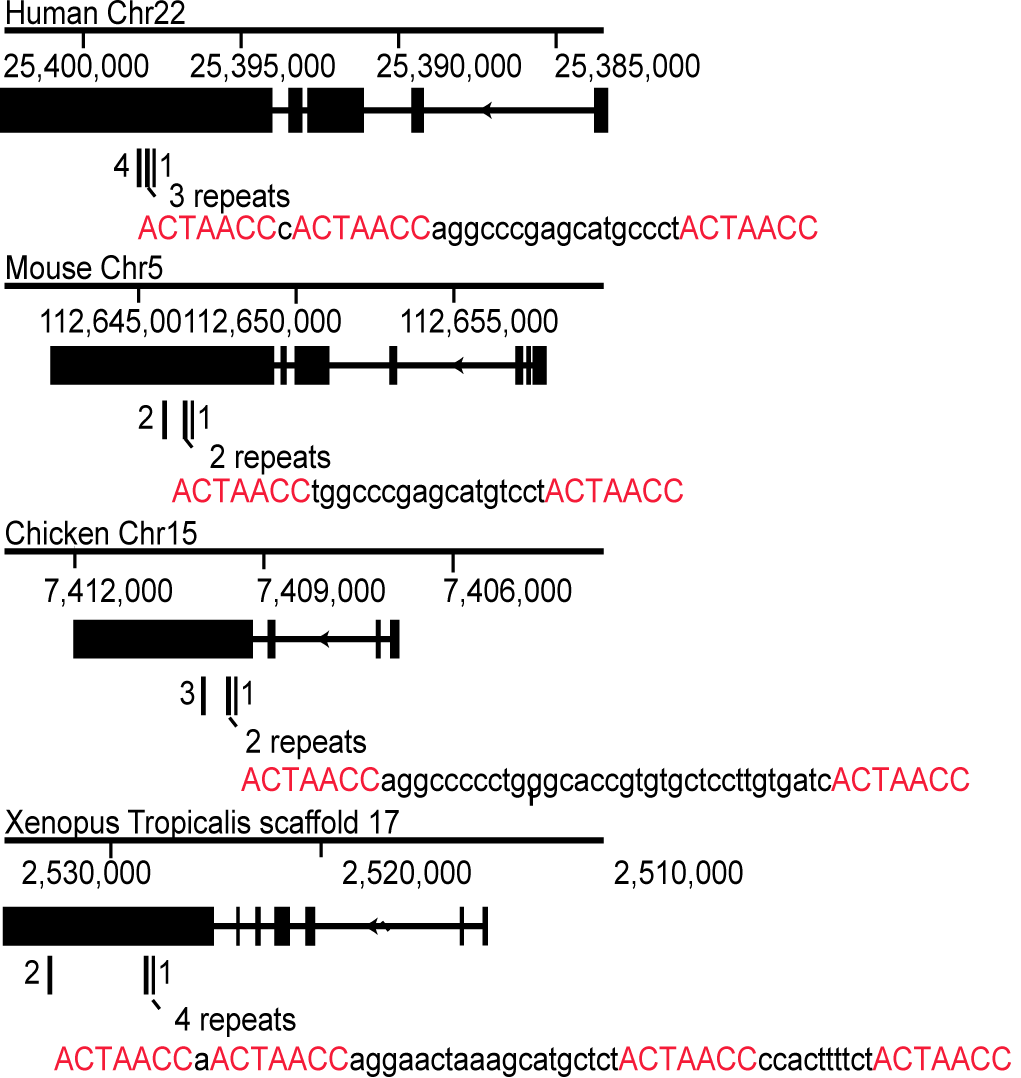
**Genomic structure and evolutionary conservation of RNCR2**. Schematic drawing showing the conservation and genomic position of RNCR2 based on the human March 2006, mouse July 2007, chicken May 2006 and Xenopus April 2005 assemblies of the UCSC Genome browser. Clusters of the conserved seven base repeat ACTAACC are shown below each RNCR2 transcript, with the sequence of one repeat cluster for each species is shown.

To determine the extent to which RNCR2 is evolutionary conserved and to identify functionally important domains, we examined genomic and cDNA sequence data from other vertebrates. RNCR2, which does not lie within 20 kb of any known protein coding gene, showed extensive sequence conservation and little diversity of splicing among mammals (Figure 1). Though conventional sequence alignment searches did not identify any nonmammalian orthologues of RNCR2 [19], a synteny-based search identified putative RNCR2 orthologues from chick and Xenopus tropicalis. Multiple clustered sequence repeats of the sequence ACUAACC were found in the largest exon of each orthologue, with anywhere from five to eight such repeats found within each transcript. A genome-wide search of human and mouse cDNA sequences did not identify any other transcripts with more than one evolutionarily conserved ACUAACC repeat (data not shown). This repeat sequence includes the ACUAAY consensus recognition site for the RNA binding protein Quaking, which regulates both RNA subcellular localization and stability [21,22], although the functional significance of this is unclear.

### Cellular expression pattern of RNCR2 in developing retina

**Figure 2:**
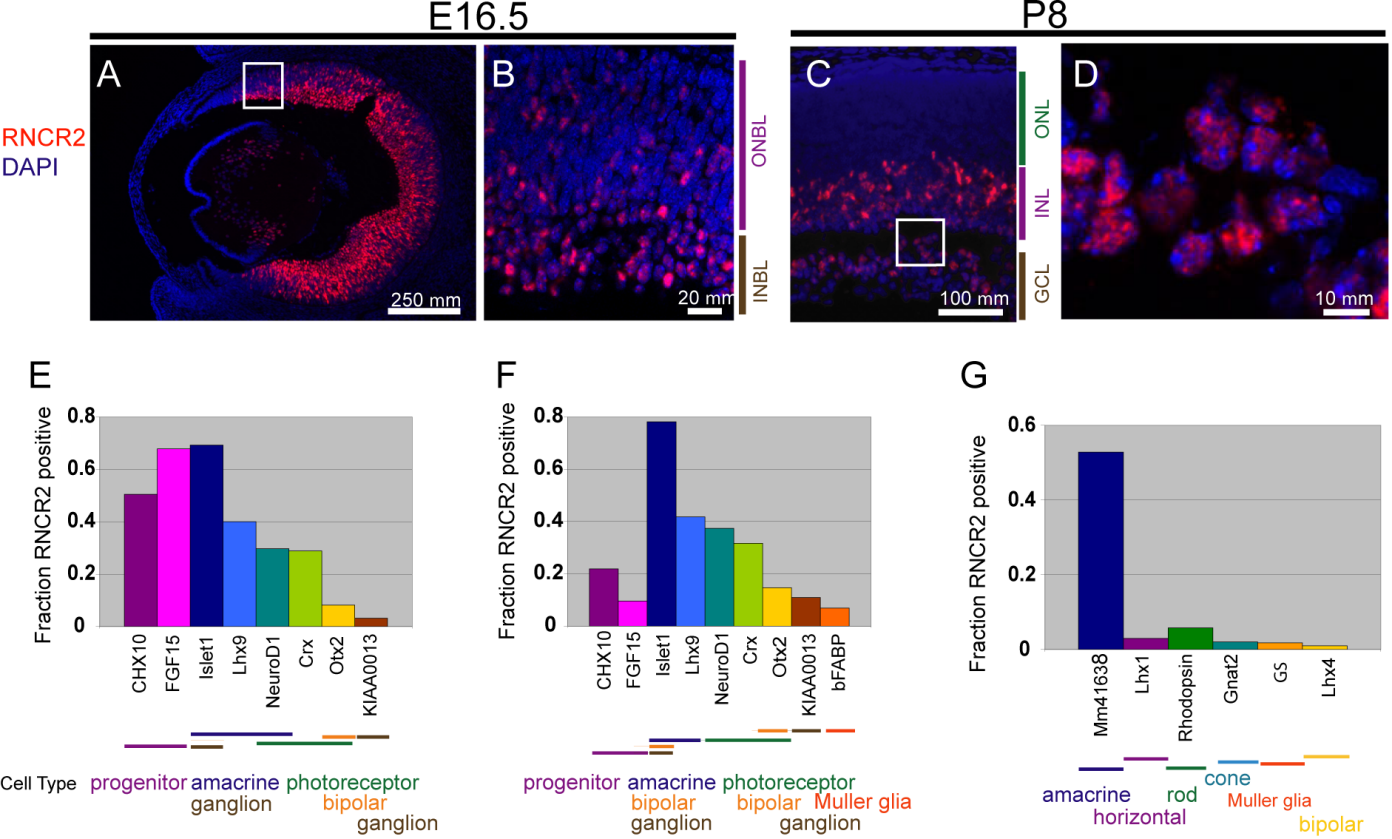
**Expression of RNCR2 in the developing retina**. Section fluorescent *in situ *hybridization (fISH) showing RNCR2 expression. RNCR2 is widely expressed in the retina at E14.5 (A), and in the INL and GCL at P8 (C). High-power magnification of E14.5 (B) and P8 (D) reveals that RNCR2 is localized to the nucleus but not associated with DNA. (E-G) RNCR2 expression examined by double dissociated-cell *in situ *hybridization at E16.5 (E), P0.5 (F), and P7 (G). Cellular expression pattern of each control probe is color coded by cell type. Some probes recognize more than one cell type. This is indicated by overlapping bars. For example Islet1 mRNA at E16.5 is present in developing amacrine and ganglion cells [34].

We next performed fluorescent *in situ *hybridization (fISH) for RNCR2. RNCR2 has a strikingly punctuate expression pattern in the developing retina at E16.5, with cells that strongly express RNCR2 intermingled with cells that express it weakly or not at all (Figure 2A). RNCR2 expression declines after the first few days postnatal, and by P8 is confined to a subset of cells in the inner nuclear layer and ganglion cell layer of the peripheral retina (Figure 2C). RNCR2 RNA is exclusively nuclear at this stage and excluded from DAPI-stained chromatin (Figure 2D), as previously reported for the embryonic retina [19]. As previously reported [18], RNCR2 is not detectably expressed in adult retina.

To determine which cells express RNCR2 in the developing retina, we used the results of a previous genome-scale analysis of gene expression in the developing mouse retina [18] to perform two-color dissociated cell fISH using multiple probes that recognized retinal cell-specific markers at different developmental stages (Additional File 1). At E16.5, we find that RNCR2 is expressed in a large fraction of cells that express the progenitor-specific markers Chx10 and Fgf15 (Figure 2E). RNCR2 is also expressed in many cells expressing the amacrine-specific marker Lhx9 and Isl1, which is expressed in developing amacrine and ganglion cells. A substantial minority of cells at this stage expressing the photoreceptor-specific marker Crx are RNCR2-positive (Figure 2E). A much smaller fraction of cells expressing the progenitor-specific markers FGF15 and Chx10 expressed RNCR2 at P0.5 than did at E16.5, while expression in amacrine and photoreceptor precursors remained prominent (Figure 2F). Finally, at P7, RNCR2 expression was restricted to a subset of amacrine cells (Figure 2G), in line with the observed expression in the inner nuclear layer (INL) and ganglion cell layer (GCL) (Figure 2C).

### Loss of function of RNCR2 inhibits amacrine cell and Müller glia differentiation in postnatal retina

**Figure 3:**
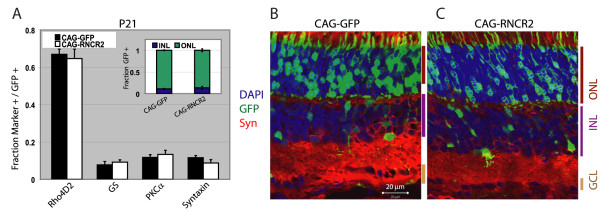
**Overexpression of RNCR2 in the developing retina**. CAG-GFP or CAG-RNCR2 construct was electroporated into P0.5 retina and harvested at P21, dissociated and stained for cell-specific markers. GFP-positive cells were counted to analyze the fraction of electroporated cells that expressed the markers in question. Error bars represent +/- SEM for at least three independent retinas. (A) CAG-RNCR2 overexpression has no effect on cell fate determination. The number of GFP-positive electroporated cells in ONL and INL were counted and compared (inset), with no difference was noted. (B-C) Section immunohistochemistry of retinas electroporated with either CAG-GFP or CAG-RNCR2 and harvested at P21 confirm no differences in cell fate or morphology of CAG-RNCR2 electroporated cells. Cell type specific markers used: rhodopsin (Rho4D2), rod photoreceptors, glutamine synthetase (GS), Müller glia; protein kinase C alpha (PKCα), rod bipolar cells; syntaxin (Syn), amacrine cells.

Given the prominent and selective expression of RNCR2 in the developing retina, we next employed *in vivo *electroporation of P0.5 mouse retinas to determine whether RNCR2 regulates retinal cell differentiation. To visualize electroporated cells, all constructs were coelectroporated with plasmids that express GFP from the same CAG promoter. For RNCR2, we assembled a 9 kb cDNA corresponding to the previously reported full-length mouse sequence [19] (Additional File 2). Overexpression of RNCR2 did not give any detectable phenotype in either section or dissociated cell immunohistochemistry (Figure 3A), and no changes in morphology or position of electroporated cells were observed at P21 (Figure 3B, C).

**Figure 4:**
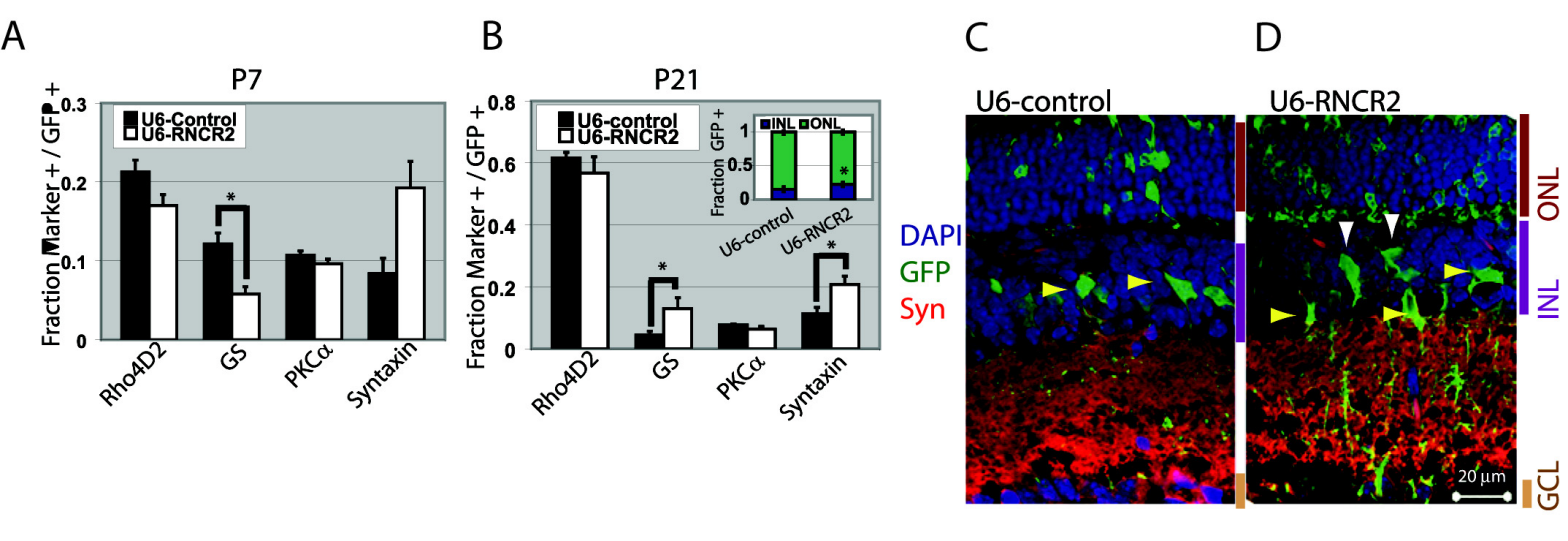
**ShRNA-mediated knockdown of RNCR2 in the developing retina**. Control shRNA or RNCR2-targeted shRNA construct was electroporated into P0.5 retina and harvested at P7 or P21, dissociated and stained for cell-specific markers. (A) RNCR2 knockdown led to a decrease in Müller glia at P7 and (B) an increase in Müller glia and amacrine cells at P21, *p < 0.05. A significant increase in GFP-positive electroporated cells in the INL was noted (B, inset). (C-D) Section immunohistochemistry of retinas electroporated with either control shRNA or RNCR2-targeted shRNA confirm an increase in Müller glia (white arrowheads) and amacrine cells (yellow arrowheads).

For knockdown analysis, we tested shRNAs for their ability to reduce expression of endogenous RNCR2 expression by *in vivo *electroporation of P0.5 retina. Reduction in RNA expression was determined by fISH analysis of P4.5 dissociated cells in conjunction with anti-GFP immunocytochemistry to identify electroporated cells (Additional File 3). A shRNA construct was identified that resulted in a >75% reduction in the number of double-labeled electroporated cells at P4.5 compared to cells electroporated with control shRNA. The shRNA-mediated knockdown of RNCR2 led to a decrease in the fraction of cells expressing the Müller glia marker glutamine synthase (GS) at P7 (p < 0.005, Figure 4A). Two weeks later at P21, however, a significant increase in the fraction of both GS-positive Müller glia (white arrowheads) and syntaxin-positive amacrine cells (yellow arrowheads, p < 0.05) was observed at P21 (Figure 4B-D). This finding was confirmed by analysis of immunostained sections of electroporated retina (Figure 4C, D).

A significant increase in the fraction of electroporated cells in the inner nuclear layer (INL) (p < 0.04), together with a corresponding decrease in the fraction of electroporated cells in the photoreceptor-containing outer nuclear layer (ONL) (Figure 4B, inset) was detected following RNCR2 knockdown, in line with the observed increase in cells expressing amacrine and Müller glial markers (Figure 4B). No change in the fraction of electroporated cells expressing the rod bipolar marker PKCα was observed following RNCR2 knockdown (Figure 4A, B). Surprisingly, however, no statistically significant reduction in the number of cells expressing the rod-specific marker Rho4D2 was observed (Figure 4B). Additionally, no increase in apoptotic cell death, as indicated by TUNEL staining, was observed following RNCR2 knockdown at P4.5. One possible explanation is that a subset of inner retinal cells may continue to express rhodopsin in addition to syntaxin and/or GS, although we have seen no evidence to support this using section immunohistochemistry (data not shown).

### Fusion of RNCR2 to an IRES-GFP sequence results in nuclear export and a dominant-negative phenotype *in vivo*

**Figure 5:**
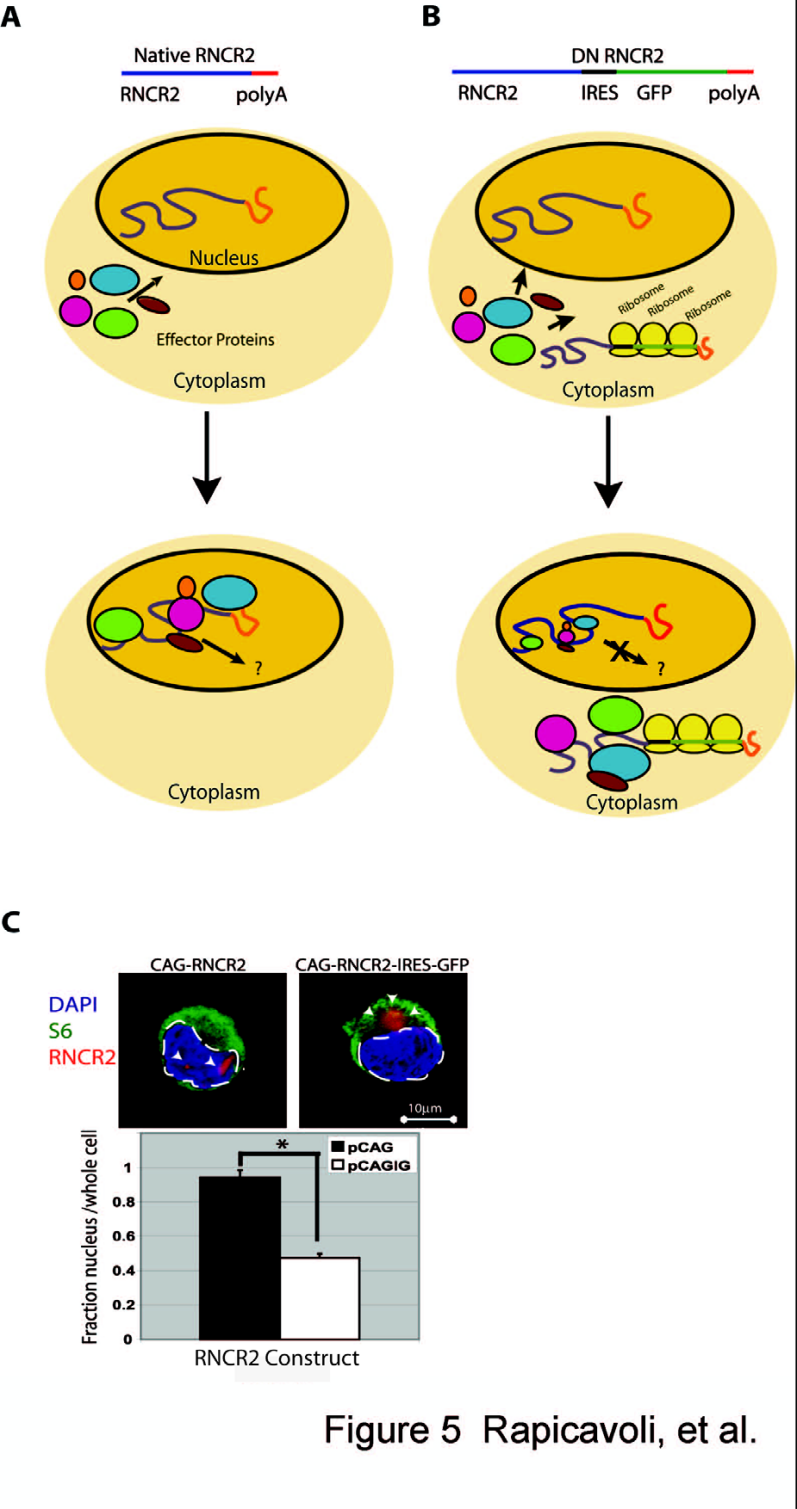
**IRES-GFP fusion constructs mislocalize RNCR2 to the cytoplasm**. (A, B) Model for a mechanism by which overexpression of IRES-GFP fusion-constructs can result in dominant-negative phenotypes in transfected cells. Fusion to the IRES-GFP sequence mislocalizes lncRNAs and associated effector proteins to the ribosome, and mislocalizes proteins that bind RNCR2 and mediate the biochemical effectors of lncRNA function away from endogenous nuclear-retained lncRNAs, effectively inhibiting their function. (C) RNCR2-IRES-GFP RNA is mislocalized from the nucleus to the cytoplasm. HeLa cells were transfected with RNCR2 or RNCR2-IRES-GFP fusion constructs and RNA location analyzed by fISH followed by immunohistochemistry against the cytoplasmic S6 ribosomal protein. White arrows indicate regions of prominent RNCR2 localization. The cell nucleus, defined by the region encompassed by DAPI-stained chromatin, is delineated by the white dashed line. The relative fraction of nuclear RNCR2 signal found within in cells transfected with CAG-RNCR2 and CAG-RNCR2-IRES-GFP is shown in the bar graph. *p < 1.5^-07^.

Throughout this study, we explored additional approaches to perturb the function of RNCR2 and to investigate the specific function of different domains of this molecule. One strategy used to generate dominant-negative forms of nuclear proteins has been to construct mutants that are sequestered in the cytoplasm, thus blocking nuclear entry of any cofactors that interact with the protein in question [23-26]. We used an analogous approach to generate dominant-negative forms of RNCR2. We adapted this strategy for analysis of RNCR2. We hypothesized that lncRNAs such as RNCR2, like proteins, must interact with cellular effector molecules to carry out their physiological functions and that mislocalization of long ncRNAs might then result in mislocalization of the associated effector molecules. Since long ncRNAs are not translated, the fusion of IRES-GFP sequences to ncRNAs would mislocalize these molecules to cytoplasmic ribosomes, and away from their normal cellular sites of action in the nucleus, titrating down the endogenous biochemical effectors of RNCR2 action and effectively resulting in a dominant-negative phenotype. Figures 5A and 5B illustrate this concept schematically. We first tested whether fusion of RNCR2 to IRES-GFP would mislocalize the RNA to the cytoplasm. Using fISH in conjunction with immunostaining for the ribosomal protein S6, we observed that full-length RNCR2-IRES-GFP constructs are mislocalized from the nucleus to the cytoplasm when expressed in HeLa cells (Figure 5C). Cells transfected with RNCR2-IRES-GFP showed GFP fluorescence, indicating that these IRES-fusion constructs are indeed targeted to the ribosome and undergo translation (data not shown).

**Figure 6:**
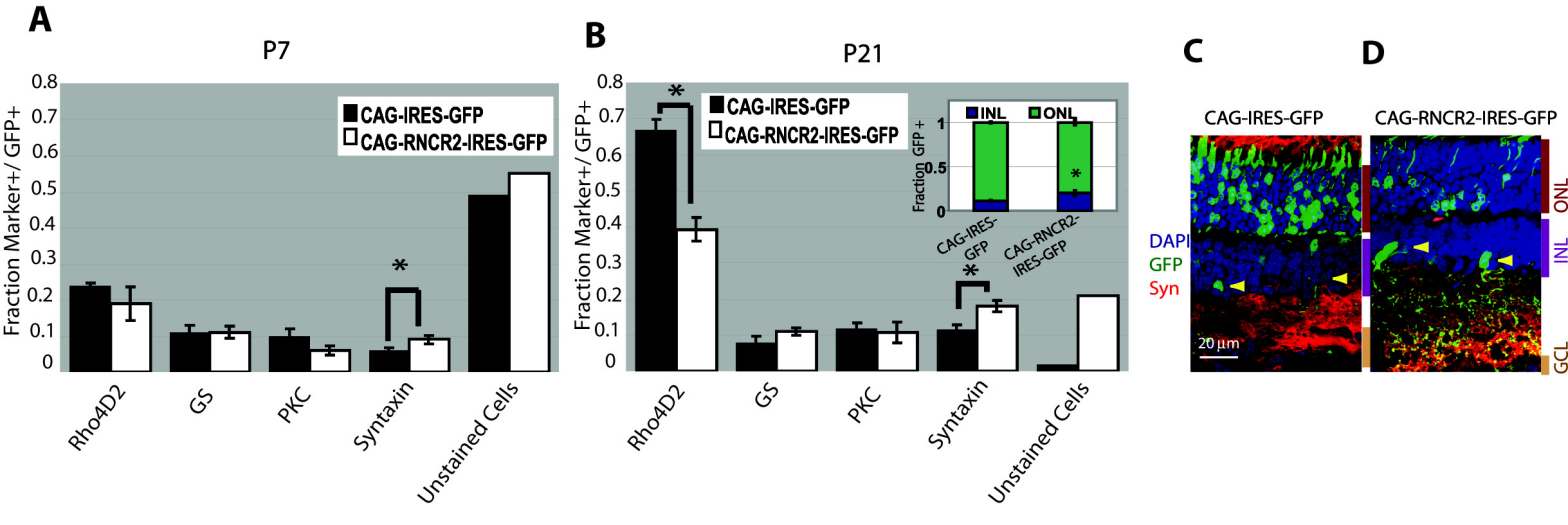
**Overexpression of RNCR2-IRES-GFP has dominant-negative effects *in vivo***. CAG-driven constructs encoding IRES-GFP or RNCR2-IRES-GFP were electroporated into P0 retina and harvested at either P7 or P21 and analyzed. (A-B) RNCR2-IRES-GFP expression results in a decrease in rhodopsin expression and an increase in the fraction of amacrine cells at P7 and P21, and an increase in the fraction of GFP-positive cells not stained by any marker tested at P21. *p < 0.02. (C-D) Analysis of electroporated retinas at P21 confirms the increase in amacrine cells (yellow arrowheads). RNCR2-IRES-GFP led to an increase in INL cells compared to IRES-GFP (B, inset).

We next tested whether electroporation of IRES-GFP fusion constructs into P0.5 retina produced phenotypes like those seen following shRNA-mediated knockdown of RNCR2. Expression of RNCR2-IRES-GFP resulted in a significant increase in the fraction of syntaxin-positive cells at P7 and P21 (p < 0.05 and p < 0.001, Figure 6A,B), as was observed with shRNA-mediated knockdown of RNCR2 at P21 (Figure 3E), and a significant increase in the fraction of cells in the INL and decrease in cells in the ONL (p < .003) (Figure 6B, inset). An increase in amacrine cells was confirmed by section immunohistochemistry of electroporated retina (yellow arrowheads, 6C-D). Furthermore, expression of RNCR2-IRES-GFP results in a substantial increase in the fraction of cells that do not express any of the four markers examined (Figure 6A,B).

Section immunohistochemistry indicates a roughly two-fold increase in the fraction of electroporated cells in the inner nuclear layer, comparable to that observed following shRNA-mediated knockdown of RNCR2 (Figure 4B), and overexpression of RNCR2-IRES-GFP further resulted in a nearly two-fold decrease in the fraction of rhodopsin-positive cells at both P7 and P21 (Figure 6A-B). Since the decrease in the fraction of rhodopsin-expressing cells was substantially greater than the observed increase in the fraction of cells in the INL, this data implies that overexpression of RNCR2-IRES-GFP induces both a shift in electroporated retinal precursors away from a rod photoreceptor fate and a reduction in rhodopsin expression levels, as implied by the dramatic increase in the fraction cells of electroporated with the RNCR2-IRES-GFP construct that are not labeled by any of the four markers tested. No increase in apoptotic cell death, as indicated by TUNEL staining, was observed following electroporation of RNCR2-IRES-GFP at P4.5 nor was any increase in staining for the cone-specific marker Gtγ2 observed at P21 (data not shown) eliminating the possibility these changes in rhodopsin expression levels are a result of apoptosis or a change in cell fate from rod photoreceptors to cone photoreceptors.

**Figure 7:**
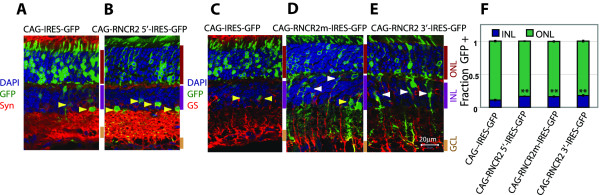
**Domain-specific RNCR2-IRES-GFP fusion constructs selectively inhibit either amacrine or Müller glia differentiation**. Dominant-negative RNCR2 constructs were electroporated *in vivo *at P0.5 and analyzed at P21. (A-B) Section immunohistochemistry reveals that RNCR2-5'-IRES-GFP overexpression led to an increase in amacrine cells (yellow arrowheads), (C-E) RNCR2-middle-IRES-GFP overexpression led to an increase in Müller glia (white arrowheads) and amacrine cells and RNCR2-3'-IRES-GFP overexpression led to an increase in Müller glia. (F) Each of the RNCR2-IRES-GFP domain-specific constructs showed an increase in INL cells compared to control **p < 0.003.

To further explore the phenotypes seen following RNCR2-IRES-GFP expression, we generated domain-specific IRES-GFP fusions corresponding to non-overlapping 2-4 kb domains of the 5', middle and 3' regions of the 9 kb RNCR2 RNA (Additional File 2), and overexpressed these in P0.5 retina using *in vivo *electroporation. Section immunohistochemistry revealed that the expression of IRES-GFP fusion constructs of the 5' domain of RNCR2 (RNCR2 5'-IRES-GFP) led to an increase in the fraction of amacrine cells, as was observed following overexpression of the full-length RNCR2-IRES-GFP construct (yellow arrowheads, Figure 7A-B). However, IRES-GFP fusion constructs of the middle 3.8 kb of RNCR2 (RNCR2m-IRES-GFP) led to an increase in both Müller glia (white arrowheads) and amacrine cells (yellow arrowheads, Figure 7C-D), while a construct including the 3' region of RNCR2 (RNCR2 3'-IRES-GFP) led to an increase in Müller glia only (white arrowheads, Figure 7E). The increase in Müller glia that is seen following overexpression of the middle and 3' IRES-GFP fusion domains mirrors that observed following shRNA-mediated knockdown of RNCR2 expression (Figure 4B). Finally, all three domain-specific IRES fusion constructs resulted in an increase in the total fraction of GFP-positive cells in the inner nuclear layer (p < 0.003, Figure 7F), reflecting the increase in amacrine cells and Müller glia like that observed following shRNA-mediated knockdown of RNCR2. We interpret these results to show that IRES-GFP fusion constructs representing distinct domains of RNCR2 possess different functions in control of retinal differentiation. Since RNCR2 shRNA results in an increase in the fraction of Muller glia but the full-length RNCR2-IRES-GFP construct does not (Figure 4B,D; 6B), these findings suggest that domain-specific IRES-GFP fusion constructs of RNCR2 may be more effective at inhibiting differentiation of certain cell types than the full-length construct. When taken together, these results closely phenocopy the changes in cell fates observed following shRNA-mediated knockdown of this RNA.

## Discussion

### RNCR2 negatively regulates development of multiple retinal cell types

Our findings demonstrate that the overexpression and knockdown of RNCR2 in the developing retina regulates the process of cell fate specification. In keeping with RNCR2's prominent and selective expression in mitotic progenitors and postmitotic retinal precursor cells, we observe that RNCR2 acts to negatively regulate the differentiation of multiple and partially overlapping cell types (Figure 5). This is in contrast to the role of Tug1, the only long ncRNA previously investigated in the context of retinal development [16], which positively regulates photoreceptor development and survival. It has recently become clear that negative regulatory interactions play a prominent and crucial role in retinal cell fate specification [27-29]. RNCR2 represents the first long ncRNA reported to perform this function.

These findings demonstrate that RNCR2 negatively regulates amacrine differentiation, as both RNCR2 knockdown and RNCR2-IRES-GFP expression result in an increase in GFP-positive cells with amacrine-like morphology, as well as in syntaxin immunoreactivity. However, it seems at first glance difficult to reconcile this finding with the fact that RNCR2 is also selectively expressed in immature amacrine cells. Several examples of a similar correlation of phenotype and gene expression have been previously reported in developing retina. One example is Pax6, which is prominently expressed in both retinal progenitors and developing amacrine cells. When Pax6 is selectively inactivated in retinal progenitors, a massive overproduction of GABAergic amacrine cells is seen [30]. It is thought that Pax6 may act in retinal progenitors to inhibit premature amacrine differentiation, but have different functions in cells already committed to the amacrine lineage. RNCR2 may play a similar role in retinal progenitor and precursor cells.

In addition to inhibiting the development of amacrine interneurons, RNCR2 also inhibits the development of Müller glia but does not affect the development of other neuronal subtypes such as bipolar interneurons. Interestingly, however, we observe that shRNA-mediated knockdown of RNCR2 results in a reduction of cells expressing the Muller marker GS at P7, in contrast to the significant increase in GS-positive cells that is observed at P21. It is possible that RNCR2 knockdown induces a delay in the differentiation of Muller glia, which initiate expression of GS shortly prior to P7. By P21, however, gliogenesis is complete and all differentiated Muller glia should be expressing detectable levels of GS. Thus, we hypothesize that these data may result from RNCR2 knockdown inducing a delay in Muller glia differentiation while nonetheless also inducing a shift in retinal progenitor cells towards a glial fate. Taken together, our findings suggest that the effects of RNCR2 loss of function on retinal cell differentiation seen in these studies are specific and do not represent a general developmental delay or inhibition of neurogenesis.

Although these findings demonstrate the functional importance of RNCR2 in retinal cell differentiation, its molecular mechanism of action still remains unclear. Unlike other nuclear-retained long ncRNAs such as Xist and Air, it shows no association with chromatin and is tightly associated with nuclear matrix [19]. It shows no obvious structural motifs, but is conserved from amphibians to mammals (Figure 1) and contains multiple putative Quaking binding sites. The biochemical mechanism of action by which RNCR2 controls retinal cell fate specification awaits further characterization.

### Mislocalization of nuclear-retained lncRNAs to the cytoplasm results in a dominant-negative phenotype *in vivo*

There have been no general strategies yet proposed for the generation of dominant-negative constructs for long ncRNAs. In this study, we demonstrate that fusion of a nuclear-retained lncRNAs to an IRES-GFP sequence can mislocalize exogenous ncRNAs to the ribosome, and produces phenotypes that largely overlap with those seen with shRNA-mediated knockdown. Indeed, the overexpression of IRES-GFP fusion constructs encoding different domains of RNCR2 variously increases the number of amacrine cells, Müller glia, or both cell types like what is seen in the RNCR2 shRNA-mediated knockdown, depending on the domain of RNCR2 that is tested. Certain novel phenotypes are observed following overexpression of RNCR2-IRES-GFP fusions that are not seen following RNCR2 knockdown, most notably a substantial reduction in the fraction of rhodopsin-expressing cells. It is possible that RNCR2 has a role in regulating expression of rhodopsin, and that this function might be inhibited more effectively by overexpression of the IRES-GFP fusion than by the RNCR2 shRNA construct. However, it is also possible that overexpression of the RNCR2-IRES-GFP fusion construct may introduce a neomorphic phenotype in developing photoreceptors that is not apparent following overexpression of RNCR2 alone (Figure 3A). Further studies will be required to resolve this question.

Many of the thousands of mammalian lncRNAs are nuclear-retained [31]. Although other nuclear-retained lncRNAs in addition to RNCR2 have been shown to be susceptible to shRNA-mediated degradation [32], conventional RNA interference approaches are in general far less successful at reducing expression of these molecules than cytoplasmic transcripts [33]. The fact that the IRES-GFP sequence mislocalizes untranslated lncRNAs from the nucleus to the ribosome may serve as a general tool for functional analysis of these molecules.

## Conclusion

We show that the long noncoding nuclear-localized RNA RNCR2 regulates retinal cell fate specification. We find that shRNA-mediated loss of function of RNCR2 increases the fraction of amacrine interneurons and Müller glia at the expense of photoreceptor cells. We further show that fusion of RNCR2 to an IRES-GFP sequence results in mislocalization of this nuclear RNA to the cytoplasm. This largely phenocopies the effects of knockdown of RNCR2, and we further use this approach to define domains of RNCR2 that inhibit amacrine and Müller glial differentiation, respectively. This IRES-GFP fusion technique provides a rapid, general method for functionally analyzing nuclear-localized noncoding RNAs *in vivo*, and can potentially allow one to readily observe more severe phenotypes than are seen using RNA interference methodology.

## Methods

### Animals

Pregnant CD1 mice were purchased from Charles River Breeding Laboratories. Animal experiments were approved by the Johns Hopkins University Institutional Animal Care and Use Committee.

### DNA Constructs

To make pCAGIG-RNCR2, the last 6535 BP of RNCR2 were amplified off BAC clone RP24-308B24 by PCR, cut with EaglI and ligated into pCAGIG RNCR2 5' digested with NotI. To make pCAG-RNCR2, RNCR2 was amplified off pCAGIG RNCR2 by PCR and cloned into pCR4 BLUNT-TOPO ((Invitrogen) per manufacture's instructions and RNCR2 was excised with NotI from pCR4 BLUNTTOPO and ligated into pCAG digested with NotI. To make pCAG-RNCR2 5'-IRES-GFP (1-2165), RNCR2 5'was excised from CF182323 with HincII and NotI and ligated into pCAG and pCAGIG digested with EcoRV and NotI. To make pCAGIG pCAG-RNCR2m-IRES-GFP (1985-5718), RNCR2 middle was amplified off RP24-308B24 by PCR and cloned into pCAG and pCAGIG digested with EcoRI. To make pCAG-RNCR2 3'-IRES-GFP' (5654-8709), RNCR2 3' was excised from BU515659 with EcoRV and NotI and ligated into pCAG and pCAGIG digested with EcoRV and NotI. ShRNA against RNCR2 was targeted against chr5:112,657,653-112,657,673 (mm9 assembly, UCSC genome browser), a site selected using Ambion's Target Finder software http://www.ambion.com/techlib/misc/siRNA_finder.html. Oligonucleotides were annealed and ligated into pSilencer 2.1-U6 hygro (Ambion). Control shRNAs cloned into pSilencer 2.1 were previously described [36].

### *In Vivo *Electroporation

*In vivo *electroporation of mouse retina was performed as described [37]. All retinas were electroporated at P0.5. Control and experimental constructs were co-electroporated with two hundred nanograms of pCAG-GFP to readily visualize electroporated cells [37]. For overexpression experiments either two micrograms of pCAG-GFP or two micrograms of pCAG-RNCR2 was injected. For shRNA mediated knockdown, two micrograms of U6-control or two micrograms of U6-RNCR2 was injected. For analysis of dominant-negative constructs, two micrograms of either pCAG-GFP or two micrograms of pCAG-RNCR2-IRES-GFP were injected.

### Cell Culture

HeLa cells were grown in DMEM supplemented with 10% FCS. Cells were transfected with Fugene6 (Roche) according to the manufacturer's instructions, and were harvested two days post-transfection.

### Immunohistochemistry

For cryosections, electroporated eyes were harvested 21 days after electroporation, fixed for 1 hour in 4% paraformaldehyde in PBS at 4°C and cryoprotected overnight in 30% sucrose in PBS at 4°C before being embedded in OCT compound (Sakura, Torrance, CA) on dry ice. 20 μm cryosections were cut on a cryostat. Retinal cryosections were immunostained as described except that sections were post fixed in 4% paraformaldehyde for 5 minutes prior to blocking [16]. Samples were photographed on a Zeiss Meta 510 confocal microscope. Electroporated cells were counted in the inner nuclear layer versus outer nuclear layer and compared for different constructs. Cell dissociation and immunostaining of electroporated retinas were preformed as described [16], except that retinas were harvested at postnatal days 7 and 21. Samples were visualized and quantified on a Zeiss Axioskop2 microscope. Nuclear DNA was visualized with DAPI counterstaining. A two-tailed Student's t-test analysis was used for statistical analysis of all cell counts in the study. At least three retinas were counted for each construct examined using either section immunohistochemistry or dissociated immunocytochemistry, with 100-300 GFP-positive cells counted for each marker tested.

Antibodies and antibody concentrations used for section immunohistochemistry are as follows: anti-glutamine synthetase (1:200, BD transduction laboratories 610518), anti-CHX10 (1:200, Abcam ab16142), anti-HPC1 (1:200, Sigma S0664), and anti-green fluorescent protein (1:1000 Invitrogen A6455). Alexa568 goat anti-mouse IgG secondary antibody (1:500 Invitrogen A11008) and Alexa488 goat anti-rabbit IgG (1:500, Invitrogen A11004).

For dissociated retinal cell immunocytochemistry the following antibodies and concentrations were used: anti-rhodopsin (Rho4D2, 1:2000, obtained from R. Molday, University of British Columbia), anti-glutamine synthetase (1:2000, BD transduction laboratories 610518), anti-protein kinase Cα (1:5000 Millipore 05-154), anti-HPC1 (1:2000, Sigma S0664), anti-green fluorescent protein (1:1000 Invitrogen A6455). Alexa568 goat anti-mouse IgG (1:1000 Invitrogen A11008), Alexa568 goat anti-guinea pig IgG (1:1000 Invitrogen A11075), Alexa 594 donkey anti-sheep IgG (1:1000 Invitrogen A11016) and Alexa488 goat anti-rabbit IgG (1:1000, Invitrogen A11004).

### *In Situ *Hybridization

For cryosections, untimed E16.5 embryos were dissected and fixed overnight in 4% paraformaldehyde in PBS at 4°C and cryoprotected overnight in 30% sucrose in PBS at 4°C before being embedded in OCT compound (Sakura, Torrance, CA) on dry ice. 20 μm cryosections were cut on a cryostat. All probe sequences used and section ISH methodology was as previously described [18] except that a Tyramide Signal Amplification system (TSA Plus, Perkin Elmer, NEL 744) combined with an antidigoxigenin-HRP antibody (1:1000, Roche) was used according to the manufacturer's directions. Sections were counterstained with DAPI.

Dissociated cell fISH was preformed as described [18] except that retinas were dissected from E16.5, P0.5, and P7 mice and the TSA Plus Cyanine3/Fluorescein kit (1:125, Perkin Elmer, NEL 753) was used for two color fISH. Samples were visualized and quantified on a Zeiss Axionskop2 microscope. Nuclear DNA was visualized with DAPI counterstaining. Cell counts were analyzed using the two-tailed Student's t-test. Dissociated cells from two to three different retinas were examined for each probe tested, and 100-300 RNCR2-positive cells were examined for colocalization with each indicated marker.

To quantify shRNA-mediated knockdown of RNCR2, retinas were dissected at P4.5 and fISH was preformed as described above using TSA Plus Cyanine3 kit (1:125, Perkin Elmer, NEL 744) followed by staining as described with rabbit anti-GFP and detection with goat anti-rabbit Alexa488. Samples were visualized on a Zeiss Axionskop2 microscope and signal intensity was quantified with Volocity 4.0 software. HeLa cell fISH followed by immunocytochemistry was performed as above except that transfected cells were trypsin digested for 5 min at 37°C before being plated and cytoplasmic ribosomes were visualized with rabbit anti-S6 ribosomal protein. A minimum of ten transfected HeLa cells were analyzed for each condition tested. Samples were photographed on a Zeiss Meta 510 confocal microscope and nuclear transcript levels were quantified using Volocity 4.0 software.

## Abbreviations

ncRNA: noncoding RNA; ncOST: noncoding opposite-strand transcript; E(number): embryonic day of gestation; P(number): postnatal age in days; shRNA; ONL: outer nuclear layer; INL: inner nuclear layer; GCL: ganglion cell layer; short hairpin RNA; IRES: internal ribosome entry site; GFP: green fluorescent protein; fISH: fluorescent *in situ *hybridization; GS: glutamine synthase; PKCα: protein kinase C alpha.

## Competing interests

The authors declare that they have no competing interests.

## Authors' contributions

N.A.R., E.M.P. and S.B. designed experiments, performed research, generated new reagents, and analyzed data. N.A.R. and S.B. wrote the paper. All authors read and approved the final manuscript.

## Supplementary Material

Additional file 1 **Dissociated dissociated-cell *in situ*hybridization**. Retinas were dissociated as described [18] at E16.5, P0.5 and P7. All cells were stained with DAPI, and probed with *in situ *probes corresponding to RNCR2 (green) and a variety of cell type-specific markers (red). At E16.5 (A-H), cells were stained with Chx10 (A), FGF15 (B), Islet1 (C), Lhx9 (D), NeuroD1 (E), Crx (F), Otx2 (G) and KIAA0013 (H), respectively. At P0.5 (I-O) dissociated cells were stained with Chx10 (I), FGF15 (J), bFABP (K), Lhx9 (L), NeuroD1 (M), Otx2 (N), and Crx (O), respectively. At P7 (P-U) cells were stained with Mm41638 (P), Lhx1 (Q), Rhodopsin (R) Gnat2 (S), GS (T), and Lhx4 (U). Click here for file

Additional file 2 **RNCR2 genomic structure and constructs used**. Genomic location of RNCR2 constructs used. Conservation is plotted in blue using the PhastCons program [35]. Click here for file

Additional file 3 **Confirmation of shRNA knockdown of endogenous RNCR2**. (A-C) A construct encoding control shRNA or shRNA targeting RNCR2 was electroporated into P0.5 retina *in vivo *and harvested at P4.5 and dissociated. Immunostaining for GFP (green) was then conducted in combination with fISH to detect RNCR2 (red). (A) GFP positive cells were quantified to analyze the amount of RNCR2 transcript that was expressed with Velocity 4.0 software. At least three retinas with 100 cells per retina were counted for each combination. Error bars represent standard error for at least three independent retinas. (A) p = 0.001. (B-C) Examples of dissociated cells are shown. Click here for file
