# Supplementary figures and images for: The long noncoding RNA RNCR2 directs mouse retinal cell specification
Source: BMC Dev Biol. 2010 May 11;10:49. doi: 10.1186/1471-213X-10-49 (PMC2876091; doi:10.1186/1471-213X-10-49)

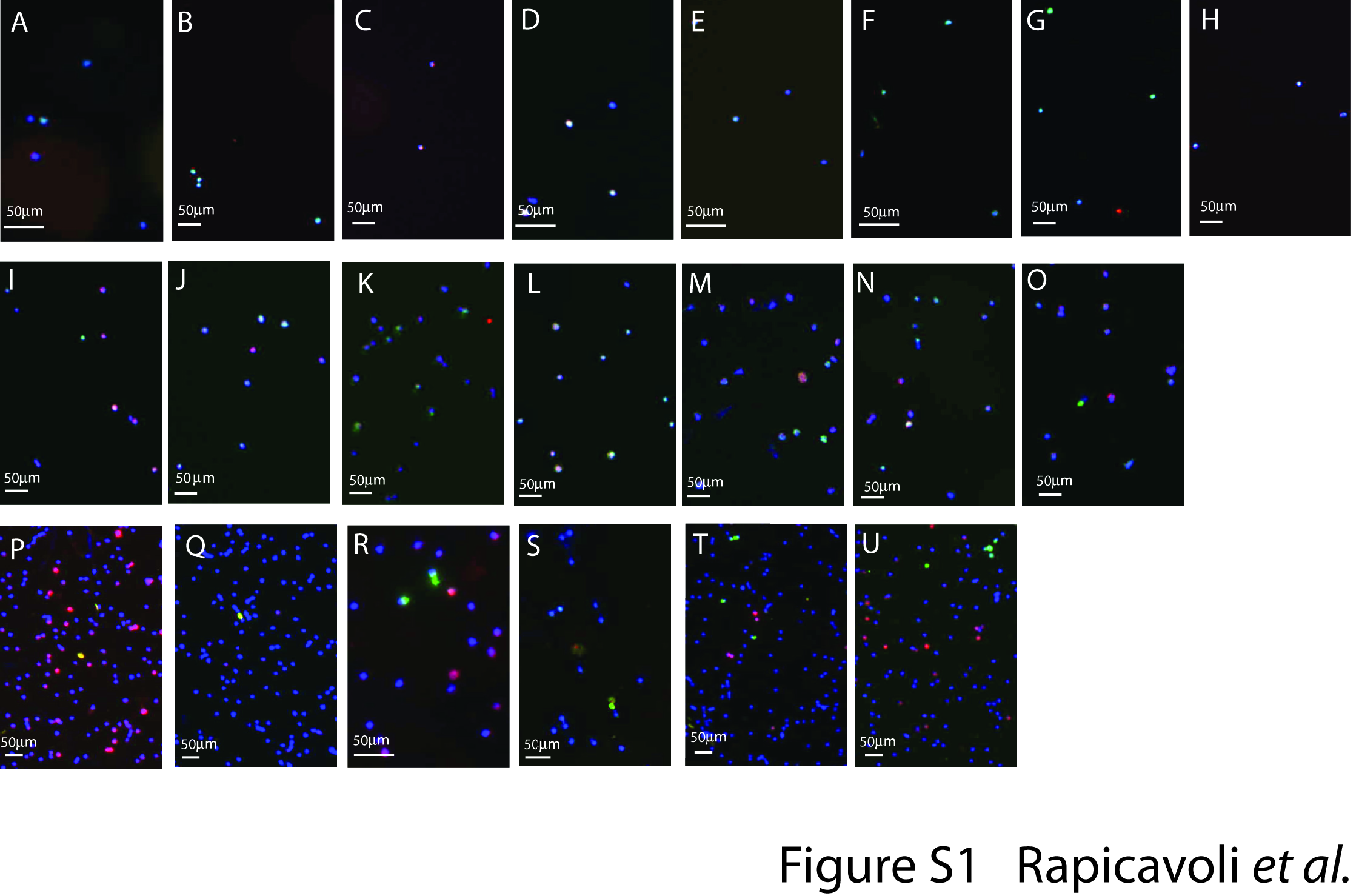

Supplement: Additional file 1 — Dissociated dissociated-cell in situhybridization. Retinas were dissociated as described [18] at E16.5, P0.5 and P7. All cells were stained with DAPI, and probed with in situ probes corresponding to RNCR2 (green) and a variety of cell type-specific markers (red). At E16.5 (A-H), cells were stained with Chx10 (A), FGF15 (B), Islet1 (C), Lhx9 (D), NeuroD1 (E), Crx (F), Otx2 (G) and KIAA0013 (H), respectively. At P0.5 (I-O) dissociated cells were stained with Chx10 (I), FGF15 (J), bFABP (K), Lhx9 (L), NeuroD1 (M), Otx2 (N), and Crx (O), respectively. At P7 (P-U) cells were stained with Mm41638 (P), Lhx1 (Q), Rhodopsin (R) Gnat2 (S), GS (T), and Lhx4 (U). [file 1471-213X-10-49-S1.TIFF]

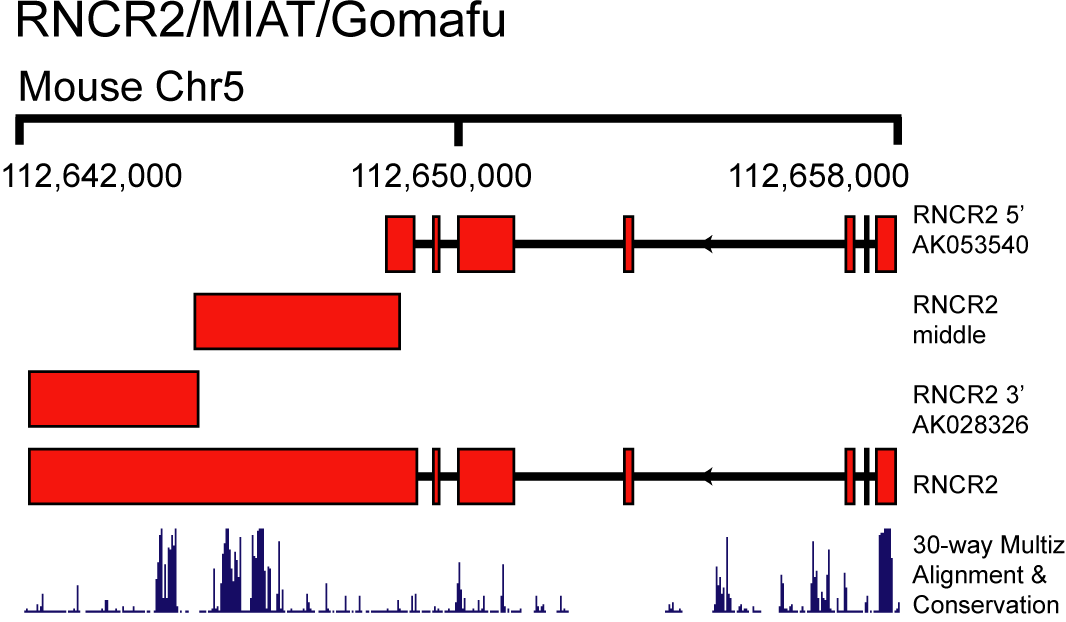

Supplement: Additional file 2 — RNCR2 genomic structure and constructs used. Genomic location of RNCR2 constructs used. Conservation is plotted in blue using the PhastCons program [35]. [file 1471-213X-10-49-S2.TIFF]

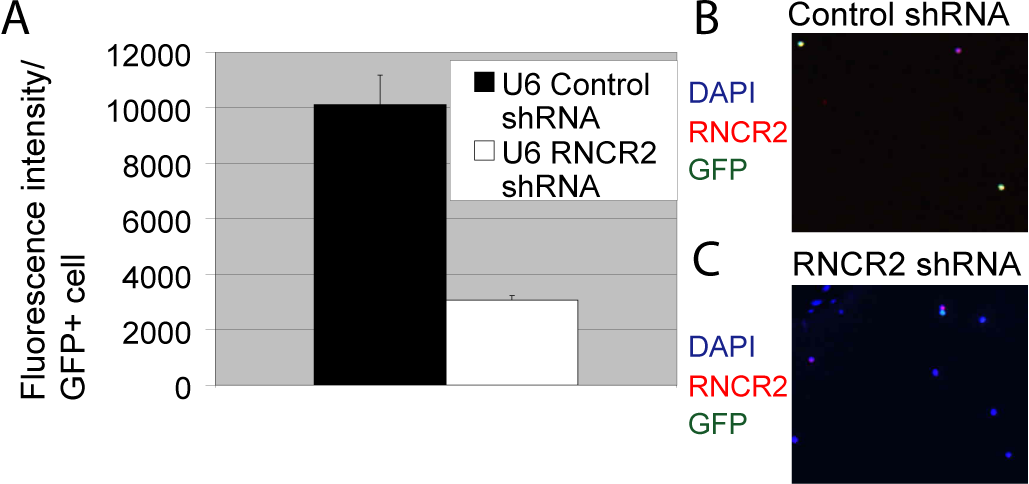

Supplement: Additional file 3 — Confirmation of shRNA knockdown of endogenous RNCR2. (A-C) A construct encoding control shRNA or shRNA targeting RNCR2 was electroporated into P0.5 retina in vivo and harvested at P4.5 and dissociated. Immunostaining for GFP (green) was then conducted in combination with fISH to detect RNCR2 (red). (A) GFP positive cells were quantified to analyze the amount of RNCR2 transcript that was expressed with Velocity 4.0 software. At least three retinas with 100 cells per retina were counted for each combination. Error bars represent standard error for at least three independent retinas. (A) p = 0.001. (B-C) Examples of dissociated cells are shown. [file 1471-213X-10-49-S3.TIFF]
